# Penetration Enhancement of Topical Formulations

**DOI:** 10.3390/pharmaceutics10020051

**Published:** 2018-04-17

**Authors:** Keng Wooi Ng

**Affiliations:** School of Pharmacy, Faculty of Medical Sciences, Newcastle University, Newcastle upon Tyne NE1 7RU, UK; keng.ng@newcastle.ac.uk; Tel: +44-1912-082343

This special issue, which is entitled “Penetration Enhancement of Topical Formulations”, presents a selection of the latest research that elucidates the challenges facing topical formulations for human skin in addition to proposing interesting solutions.

Topical drug delivery dates back to ancient times and has had a long history of continued development and use. In the distant past, our forefathers must have realized that applying an antidote directly to the site of a localized medical complaint, such as pain, often was an effective way of alleviating the complaint. This mode of administering medications has long been employed in traditional medicine across various cultures [1]. Topical drug delivery, as this route of drug administration is now referred to, is mainstream in modern medicine, especially as over-the-counter formulations.

The success of topical drug delivery depends on our ability to effectively overcome biological barriers. The skin is probably the most studied of such barriers. The main function of the skin is to limit the exchange of substances between the body and the environment. The skin barrier makes drug penetration a primary challenge in ensuring the efficacy of topical drug delivery in the skin. The general principles governing drug penetration in the skin have been established (see reference [2] for review). In summary, the stratum corneum (the outermost skin layer that is cornified) is the rate-limiting barrier preventing dermal drug penetration. In other words, the rate at which a drug diffuses across the stratum corneum determines its overall rate of dermal penetration and permeation. For this reason, the scientific literature often addresses topical (local) and transdermal (systemic) drug delivery together as they share the same challenge in terms of traversing the skin barrier. By virtue of its structure and biochemical composition, the stratum corneum is selectively permeable. Generally speaking, small and moderately lipophilic molecules (molecular weight <500 Da and log *P* of 1–4) are likely to penetrate the skin well. Other drugs that do not possess these physicochemical properties usually require a suitable penetration enhancement strategy to penetrate the skin. Such penetration enhancement strategies may include physical (e.g., microneedles and iontophoresis [3]) or chemical [4,5] penetration enhancers that either diminish the barrier properties of the skin or actively drive the movement of drugs across the skin with the input of external energy. Penetration enhancers have sometimes been used synergistically [6,7].

One study by Martin et al. [8], which is reported in this special issue, exemplifies a chemical penetration enhancement strategy that is being deployed to improve topical drug delivery. They compounded and evaluated a range of topical gabapentin formulations, including hydrogels and creams, for the relief of neuropathic pain. Ethanol, dimethyl sulfoxide, dimethyl isosorbide, isopropyl myristate and propylene glycol were investigated as chemical penetration enhancers. The skin penetration of gabapentin was assessed in an ex vivo human skin model. They showed that while ethanol and dimethyl sulfoxide enhanced the skin penetration of gabapentin from Carbopol^®^ hydrogels, gabapentin compounded with the proprietary Lipoderm^®^ base presented the most clinically relevant formulation.

On the other hand, Adb et al. [9] adopted a different approach for solving a different problem. They were interested in the targeted delivery of minoxidil to hair follicles. Topical minoxidil was used to treat a certain type of hair loss (androgenetic alopecia) [10]. The vasodilator minoxidil can also lower blood pressure if absorbed systemically [11]. As far as treating hair loss is concerned, the drug appears to act directly on the hair follicle [12]. Thus, topical delivery of minoxidil to the scalp is both intuitive and rational, as it delivers the drug directly to the site of action in order to minimize systemic effects. However, accurate targeting of hair follicles is challenging. The authors formulated minoxidil as nanoemulsions and examined its dermal uptake via the stratum corneum and hair follicles. Two well-established chemical penetration enhancers, oleic acid and eucalyptol, were incorporated separately into the nanoemulsions. Their results showed significant but different minoxidil retention in the skin layers and hair follicles. Their work is also reported in this special issue.

However, we are reminded by Montenegro et al. [13] that enhanced skin penetration may not always be a desirable outcome. When it comes to sunscreen safety, the aim is to limit skin penetration of chemicals from the topical formulations to avoid toxicity. The authors formulated a series of sunscreens containing the ultraviolet (UV) filters, octylmethoxycinnamate (OMC) and butylmethoxydibenzoylmethane (BMBM), as emulsions or oily lotions. After this, they measured the skin permeation of each UV filter following single or repeated administrations in vitro. They found that the composition of the vehicle and dosing regimen modulated the dermal uptake of the UV filters. Considering that the topical formulations for human skin often share common excipients, this research highlights the need to tailor each formulation to the desired application and to carefully select excipients.

The articles featured in this special issue are by no means comprehensive in their coverage of a very broad topic, but each has covered different aspects of the topic well. However, it should be said that the concept of topical drug delivery is not confined to the skin. The insights offered in this special issue should benefit both colleagues interested in topical formulations for the skin and others also working with other biological barriers.
